# Elevated Vitamin D Receptor Levels in Genetic Hypercalciuric Stone-Forming Rats Are Associated With Downregulation of Snail

**DOI:** 10.1359/jbmr.091010

**Published:** 2009-10-12

**Authors:** Shaochun Bai, Hongwei Wang, Jikun Shen, Randal Zhou, David A Bushinsky, Murray J Favus

**Affiliations:** 1 Section of Endocrinology and Metabolism, The University of Chicago Pritzker School of MedicineChicago, IL, USA; 2 Department of Medicine, University of Rochester School of MedicineRochester, New York, USA

**Keywords:** GHS rats, vitamin D receptor, hypercalciuria, intestine, kidney, Snail

## Abstract

Patients with idiopathic hypercalciuria (IH) and genetic hypercalciuric stone-forming (GHS) rats, an animal model of IH, are both characterized by normal serum Ca, hypercalciuria, Ca nephrolithiasis, reduced renal Ca reabsorption, and increased bone resorption. Serum 1,25-dihydroxyvitamin D [1,25(OH)_2_D] levels are elevated or normal in IH and are normal in GHS rats. In GHS rats, vitamin D receptor (VDR) protein levels are elevated in intestinal, kidney, and bone cells, and in IH, peripheral blood monocyte VDR levels are high. The high VDR is thought to amplify the target-tissue actions of normal circulating 1,25(OH)_2_D levels to increase Ca transport. The aim of this study was to elucidate the molecular mechanisms whereby *Snail* may contribute to the high VDR levels in GHS rats. In the study, *Snail* gene expression and protein levels were lower in GHS rat tissues and inversely correlated with *VDR* gene expression and protein levels in intestine and kidney cells. In human kidney and colon cell lines, ChIP assays revealed endogenous Snail binding close to specific E-box sequences within the human *VDR* promoter region, whereas only one E-box specifically bound Snail in the rat promoter. Snail binding to rat *VDR* promoter E-box regions was reduced in GHS compared with normal control intestine and was accompanied by hyperacetylation of histone H_3_. These results provide evidence that elevated VDR in GHS rats likely occurs because of derepression resulting from reduced Snail binding to the *VDR* promoter and hyperacetylation of histone H_3_. © 2010 American Society for Bone and Mineral Research.

## Introduction

Idiopathic hypercalciuria (IH) is found in 5% to 7% of the adult population and is the most common risk factor for calcium (Ca) oxalate (Ox) nephrolithiasis.(1) Hypercalciuria is thought to play an important role in the pathogenesis of CaOx stone formation through increased urine supersaturation with respect to Ca and Ox.(1–4) The disease is characterized by high intestinal Ca absorption and bone resorption and decreased renal Ca reabsorption resulting in hypercalciuria with normal serum Ca, normal or elevated serum 1,25-dihydroxyvitamin D [1,25(OH)_2_D], normal or low serum phosphate, and low bone mass.(1,4–10) Serum parathyroid hormone (PTH) is normal in the vast majority of patients.(1) The familial pattern of IH is consistent with a polygenic mode of inheritance.(11–13) Candidate genes involved in the pathogenesis of IH have been identified in only a small number of families.(12,13) The rat homologue of human IH, the genetic hypercalciuric stone-forming (GHS) rat, has a phenotype that exhibits many features with IH,(14) including normal serum Ca,(15) increased intestinal Ca absorption(16) and bone resorption,(17) decreased renal Ca reabsorption,(18) and normal 1,25(OH)_2_D serum levels,(15) in addition to a polygenic mode of inheritance. Most kidney stones are composed of Ca salts owing to supersaturation of the urine with respect to Ca and the dietary content of anion species such as phosphate and Ox.(19–25)

All the changes in intestine, kidney, and bone Ca transport in IH may be reproduced by the administration of small doses of 1,25(OH)_2_D_3_ to healthy human volunteers.(26,27) Elevated serum 1,25(OH)_2_D levels may account for the phenotype in 40% to 60% of IH patients.(6–8,28,29) However, the remaining patients have normal serum 1,25(OH)_2_D levels and increased Ca transport rates in intestine, kidney, and bone and are indistinguishable from patients with elevated serum 1,25(OH)_2_D levels.(6,28,30) GHS rats share similarities with IH patients with normal serum 1,25(OH)_2_D levels in that elevated intestinal, kidney, and bone VDR protein levels are found in GHS rats,(31) and high VDR levels have been found in the peripheral blood monocytes (PBMs) of male IH stone-formers.(32)

We have postulated that elevated tissue VDR may amplify the biologic actions of normal circulating 1,25(OH)_2_D levels and create hypercalciuria through increased intestinal Ca absorption and bone resorption. 1,25(OH)_2_D increases *CaR* gene expression and may decrease renal tubule Ca reabsorption through activation of CaR.(33–35) Early generations of GHS rats had elevated duodenal and kidney VDR levels(17,20,31) owing in part to prolongation of the half-life of the VDR protein.(31) *VDR* gene expression by Northern blot analysis was normal or low.(31) In subsequent generations of GHS rats, hypercalciuria has become more intense, and VDR levels and rates of gene expression are increased in intestine and kidney.(36) Investigations into the causes of elevated VDR in GHS rats have revealed increased transcription rates and prolonged turnover with no difference in the duodenal *VDR* mRNA sequence.(31)

In this study, the mechanism of upregulation of *VDR* in GHS rats was explored for evidence of cis- and trans-acting regulation. The transcription factor Snail, a negative regulator of VDR in human colon cancer cells,(37) was investigated for a trans-regulatory role in *VDR* expression. Snail is a zinc-finger transcription factor expressed in migratory processes during embryonic development(38,39) and has been implicated in the development of metastatic cancer through downregulation of *VDR* and the conversion of an epithelial to a mesodermal phenotype.(40) Homozygous *Snail* gene knockout (*Snail*^–/–^) in mice is lethal early in embryogenesis,(41) whereas heterozygous (*Snail*^+/–^) mice appear normal. No information on Snail involvement in mineral homeostasis is available, and Snail regulation of *VDR* in normal physiology or in the pathophysiology of benign conditions has not been described. To investigate the potential role of Snail in the pathogenesis of the high VDR in GHS rats, we measured *Snail* gene expression and protein levels in relation to VDR and the binding of Snail to specific sites in the rat *VDR* promoter regions, as well as the status of histone H_3_ modification within the proximal *VDR* promoter region in GHS rats.(42,43)

## Materials and Methods

### Animals

The colony of GHS rats was created by the selective breeding of male and female Sprague-Dawley rats (S-D; Harlan, Inc., Indianapolis, IN, USA) with the highest 24 hour urine excretions. Beyond the thirtieth generation, GHS rats have consistently excreted 8 to 10 times the level of urine calcium of wild-type normocalciuric (NC) S-D rats.(15) GHS rats raised at the University of Rochester were shipped to The University of Chicago at age 7 weeks. NC S-D rats purchased from Harlan, Inc., were matched for age and body weight to the GHS rats. All animal experiments were approved by The University of Chicago Institutional Animal Care and Use Committee.

### Cell lines

Three cell lines—HEK293 (human embryonic kidney) cells, SW480 (colon cancer) cells, and DLD1 (colon cancer) cells—were obtained from American Type Culture Collection (Manassas, VA, USA) and maintained in Dulbecco's modified Eagle's medium containing 10% fetal bovine serum, 50 µg/mL penicillin, 0.25 µg/mL streptomycin, and 2 mM l-glutamine at 37°C under 5% CO_2_.

### Experimental design

Male GHS and NC rats were fed normal chow containing 1.2% Ca, 0.24% Mg, 1.0% P, and 0.55 µg vitamin D_3_ per gram of rat chow. After an overnight fast, rats were placed under deep general anesthesia and exsanguinated via the abdominal aorta. Tissues were harvested, and a portion of each tissue was either rapidly added to RNeasy (Qiagen, Inc., Valencia, CA, USA) for RNA preservation and then stored at −80°C or rinsed in ice-cold PBS buffer, homogenized on ice, and then stored for subsequent Western blot and protein measurements.

### Antibodies and vectors

Monoclonal anti-VDR antibody was purchased from Chemicon, Inc. (Temecula, CA, USA). β-actin-specific antibody was purchased from Sigma-Aldrich, Inc. (St. Louis, MO, USA). Anti-Snail antibody used for Western blot, electrophoretic mobility gel shift (EMSA), and chromatin immunoprecipitation (ChIP) assays was obtained from Abcam Plc (Cambridge, MA, USA). Secondary anti-mouse and anti-rabbit horseradish peroxidase (HRP)–linked antibodies were purchased from GE Healthcare UK, Ltd. (Amersham, England). HRP-labeled anti-rat IgG was purchased from Kirkegaard & Perry Laboratories (Gaithersburg, MD, USA). Anti-acetylated H_3_ antibodies were purchased from Upstate Biotechnology (Lake Placid, NY, USA), and normal IgG was purchased from Santa Cruz Biotechnology, Inc. (Santa Cruz, CA, USA). pcDNA3 Vector was purchased from Invitrogen Corp. (Carlsbad, CA, USA). pcDNA3-*Snail* was a generous gift from Dr Amparo Cano (Institute de Invesgaciones biomedicas UAM/CSIC, Madrid, Spain).

### RNA isolation and real-time PCR

Renal and intestinal total RNA were isolated using RNeasy mini kit (Qiagen, Inc., Valencia, CA, USA) according to the manufacture's protocol. Total RNA from cell lines was extracted using Trizol (Invitrogen). First-strand cDNA synthesis was performed with an oligo dT primer using a cDNA synthesis kit (TaqMan Reverse Transcription Reagents, Applied Biosystems, Foster City, CA, USA). Gene-specific oligonucleotides used for real-time reverse-transcriptase polymerase chain reaction (RT-PCR) were designed using Primer Premier V. Blast searches were used to ensure that primers were specific for each individual gene. Real-time PCR reactions were performed using SYBR Green PCR Master Mix (Applied Biosystems) and an ABI PRISM 770 apparatus (Perkin Elmer, Applied Biosystems). Thermocycling was done in a final volume of 25 µL containing 60 ng of cDNA sample, 800 nM of each of the forward and reverse primers. The PCR was performed as the following program: 95°C for 10 minutes, 40 cycles of 95°C for 30 seconds, 65°C for 20 seconds, and 72°C for 30 seconds. The relative amount of each sample was calculated from a standard curve after employing the fit-point algorithm for quantification. A relative standard curve was generated from pooled RNA of NC and GHS samples. For accurate relative quantitation, the dilution series from which the standard curve was generated was carefully prepared using dilution factors of 1-, 3-, 9-, 27-, and 81-fold. The expression value was normalized to GAPDH or β-actin. A relative gene expression was determined by assigning the adult rat groups fed a normal calcium diet (NCD) a relative value of 1.0, with unknown values relative to this control. Primer sequences are listed in Table 1.

**Table 1:** Primers Used for Real-Time PCR

Primer	Forward Primer	Reverse Primer
*Rat VDR*	GCCCCTCATAAAGTTCCAGGTG	GGATAGGCGGTCCTGAATGG
*Rat Snail*	CTGGGCGCTCTGAAGATGCA	GGAGCAGCCAGACTCTTGGTGT
*Rat GAPDH*	GCCAGCCTCGTCTCATAGACA	AGAGAAGGCAGCCCTGGTAAC
*Human VDR*	TCCTCCTGCTCAGATCACTG	AGGGTCACAGAA GGGTCATC
*Human Snail*	CCCAATCGGAAGCCTAACTA	GGCTGCTGGAAGGTAAACTCTC
*Human β-actin*	TGGACTTCGAGCAAGAGATG	GAAGGAAGGCTGGAAGAGTG

### DNA preparation and sequencing reaction

Genomic DNA was purified using the Puregene DNA purification kit (Gentra Systems, Inc., Minneapolis, MN, USA). For SNP discovery, PCR was used to amplify the approximate 7.3 kb fragment containing the promoter, all exons, and the exon/intron boundary regions. Primers were designed according to the reference sequence AF288738 from GenBank using Primer Premier Version 5.00 (Premier Biosoft International, Palo Alto, CA, USA) (Table 2). PCR was carried out using 1 unit of Hotstar Taq polymerase (Qiagen, Inc.) with 4 mM Mg^2+^, 20% Q-solution, and 20 ng DNA in 40 µL final volume. A touchdown thermal cycling protocol was used for all amplification: 98°C for 10 minutes for denaturation and activation of DNA polymerase followed by 8 cycles of denaturation at 98°C for 20 seconds, annealing at 68°C for 20 seconds and 1°C decreasing per cycle, and extension at 72°C for 40 seconds. After touchdown, another 35 cycles were performed at 98°C for 20 seconds, 59°C for 20 seconds, and 72°C for 40 seconds. PCR products were purified with Qiaquick PCR purification Kit (Qiagen, Inc.), followed by direct sequencing from both ends using Bigdye Terminator Kit Version 3.0 (Applied Biosystems). Sequencing reaction products were precipitated and then separated on ABI-3700 capillary sequencer (Applied Biosystems). The GHS *VDR* sequences were compared with the NC *VDR* sequences using blast 2 (http://www.ncbi.nlm.nih.gov/blast/bl2seq/wblast2.cgi).Protein extraction and Western blot

**Table 2:** Primers Used for Sequencing GHS and NC Rat VDR Exons, Intron/Exon Boundary Regions, and Proximal Promoter

Primer	Forward Primer	Reverse Primer
1	CCAATCGTTCCCTTTCTTACTCC	GGTCTCCAAGGCGACAGTGCA
2	CAACCACGCCCACTAGGTTCAGT	AACGAGACGCAATTAGCCAGGAA
3	AGACGGGAATGGAAGTTGGAGT	TTGGTTTCTTGTAGGGACAGTCATC
4	GAGGTGGAGAACTGAGTGATTTGC	AATCAGTTTGGAGTATGTTGGTGCT
5	CAGGAGGAAATCTCAACAGCC	TCCAGAGAATTCCTTTCAGACTC
6	CCGAGGAAAGAAACAAACGCT	CAAGGCGACAGTGCAGTGGT
7	AATCATCCCTTGTCCATCAAAC	CGAGGTCCACGGTCTCCTCTA
8	ATGCCCTTCTTCTGCCTGAT	GCTTGCCCTCTGCCTTGA
9	GCCCTGTTGCTTGCTGTA	CCAAAGAACTGTCACCCACT
10	ACCTGCTGCATTATATGGGCTCTCT	TGGTGGCTGTCGTCGTCTCAAC
11	CTCTTTCCTTTAGCCTCAGGAC	GAAAACCCAGTGTTCTCCATG
12	GGTTCTGGTCAGGCACTCTG	GGAAAAGGCAGTCACTATTGGA
13	TGCAAACCCAGCAAAGTGAA	CGGTTCAGTCCTGCCCAAA
14	AGGATGAGGCAGACAGACAGAG	GAAGAAGTAGGGTGAGGTGGG
15	TTTCCAATGGTGGTTTCTAGG	CACAGTGGTGCCTGGGTAG

Tissue was homogenized in cold lysis 250 buffer (50 mM Tris/HCl, pH 7.4, 250 mM NaCl, 5 mM EDTA, 0.1% Nonidet P-40, 50 mM NaF, 1 mM PMSF, 1 µg/mL leupeptin, and 1 µg/mL antipain) and then chilled on ice for 30 minutes. The mix was vortexed at full-speed for 20 seconds every 5 minutes and then centrifuged for 15 minutes at 14,000 × *g* at 4°C. The supernatant was removed and stored at −80°C. Nuclear extracts were isolated from cell lines using the NE-PER kit (Pierce Biotechnology, Inc., Rockford, IL, USA). Protein concentration was determined using the Bio-Rad protein assay (Bio-Rad Laboratories, Inc., Hercules, CA, USA). Total protein aliquots of 20 µg were denatured in 6 × SDS sample buffer (7 mL 4 × Tris/HCl, SDS, pH 6.8, 3.0 mL of glycerol, 1g of SDS, 0.93 g of dithiothreitol, and 1.2 mg of bromphenol blue in 10 mL of distilled deionized H_2_O) and then loaded onto SDS denaturing discontinuous gels. The protein was transferred onto polyvinylidene difluoride membranes (Immobilon-P, Millipore Corp., Bedford, MA, USA) by electroblotting at 90 V for 1 hour. The membranes then were blocked with 5% nonfat dried milk in TBS that contained 0.1% Tween-20 (TBS-T). The primary antibody was diluted with 3% nonfat dried milk in TBS-T. Then the membrane was incubated with the diluted primary antibody at room temperature for 1 hour. Membranes were washed with TBS-T and incubated with HRP-conjugated anti-rat IgG for 1 hour at room temperature. Band intensities were developed using the ECL Chemiluminescence System (Amersham Life Science, Buckinghamshire, UK). The blots were quantified by scanning using One-Scan 1D Gel Analysis software (Scanalytics, Inc., Fairfax, VA, USA).

### EMSA assay

Human proximal tubule–derived HEK293 cells were transfected with pcDNA3 and pcDNA-*Snail* using Lipofectamine and Lipofectamine 2000 Transfection Reagent (Invitrogen). After 48 hours of transfection, nuclear extracts were isolated using the NE-PER (Pierce Biotechnology), and Snail protein was determined by Western blot.

In the EMSA assay, the double-strand oligonucleotides probes were labeled with biotin. Nonlabeled oligonucleotides were used as competitors for biotin-labeled oligonucleotide binding. The sequences flanking the E-boxes in the rat *VDR* promoter region are for E-box 1, 5'-GGTCAACTCAGGTACGGGTGACACAC-3'; for E-box 2, 5'-ACGGGTGACACACCTGGCGGAGGCGT-3'; and for E-box 3, 5'-GGGGCGGGGCCAGGTGCTGAGCAGTC-3'. Briefly, 20 µL of binding reaction containing 2 µg of nuclear extract was prepared according to the kit instruction manual and incubated at room temperature for 20 minutes. Complexes were resolved on 4% acrylamide gels (29:1 acrylamide:bisacrylamide). When electrophoresis was completed, binding reactions were electrophoretically transferred to a nylon membrane. The transferred DNA then was cross-linked to the nylon membrane, and the biotin-labeled DNA-protein complex was detected by chemiluminescence kit (Pierce Biotechnology). For antibody inhibition of protein-DNA interaction, anti-Snail antibody was added to the binding reactions prior to electrophoresis and incubated for 20 minutes at room temperature. The reaction mix then was resolved and transferred as described earlier.

### ChIP Assay

ChIP assays were performed on DNA isolated from HEK293, SW480, and DLD1 cell lines and from frozen intestine and kidney tissues as described previously(44) with some modifications. Using the cell lines, cells were cross-linked by adding 270 µL formaldehyde into 10 mL of medium for 10 minutes and then stopped by addition of glycine at a final concentration of 0.125 M. Cell lysates then were sonicated into 200 to 1000 bp DNA fragments. Anti-Snail antibody was used to immunoprecipitate the DNA-protein complex, and IgG alone was used as a negative control. After overnight incubation with antibody, immunocomplexes were collected using 80 µL of protein A agarose beads in a slurry (Upstate Biotechnology, Lake Placid, NY, USA). The agarose beads were washed twice with the following buffers (Upstate Biotechnology): low-salt wash buffer, high-salt wash buffer, LiCl wash buffer, and TE buffer. DNA was eluted with 1% SDS and 0.1 M NaHCO_3_ elution buffer, subjected to reverse cross-linking, proteinase digestion, and purification using a commercial kit (Qiagen, Inc.). Primers used to amplify the human *VDR* promoter region are 5'-CTGCAGCAGTAACAGGTTGG-3' and 5'-GCTTCAGCCTGTGTTAATCG-3'.

For the tissue ChIP assay, frozen kidney and intestine tissues from GHS and NC rats were thawed at room temperature, cut into 1 mm slices, and crossed-linked in 1.5% formaldehyde for 15 minutes. Tissues then were homogenized with Tissuemiser (Fisher Scientific, Pittsburgh, PA, USA), and the cell suspension was centrifuged at 14,000 rpm for 10 minutes. SDS lysis buffer was added to the pellets, and they were processed as described earlier except that antibodies against acetylated H_3_ were used. The primers that amplify the rat *VDR* promoter region are 5'-CCACCCATAGTTCCAGGTCT-3' and 5'-CGGACTCCACTGGTTAGGAT-3'.

### Immunostaining assay

Small intestine and kidney tissues of GHS rats were harvested, embedded in Tissue-Tek OCT (Sakura Finetek USA, Torrance, CA, USA), and snap frozen with liquid nitrogen. Then 5 µm sections were cut with a cryostat. An indirect double-fluorescence method was applied to detect the expression patterns of VDR and Snail in intestinal mucosal epithelial crypt and villus cells and in kidney segments from the loop of Henle and distal convoluted tubule regions of the nephron. In brief, intestinal and kidney slices were fixed with 4% paraformaldehyde and blocked with 5% bovine serum albumin (BSA) to eliminate nonspecific staining. For intestinal crypt and villus staining, sections were sequentially incubated with mixed rat anti-VDR monoclonal antibody (1:100; Millipore Corp., Bedford, MA, USA) plus rabbit anti-Snail polyclonal (1:100; Abcam Plc, Cambridge, MA), biotinylated mouse anti-rat IgG2b (4 µg/mL; BD Pharmingen, San Diego, CA, USA), fluorescein isothiocyanate (FITC)–conjugated streptavidin (1:100; BD Pharmingen), and then Texas red conjugated goat anti-rabbit IgG (1:100; Jackson ImmunoResearch Labs, West Grove, PA, USA). For staining of loop of Henle and distal convoluted tubule sections, rabbit anti-human VDR polyclonal antibody was obtained from Abcam Plc and used at a dilution of 1:200. Goat anti-human Snail polyclonal antibody was obtained from Santa Cruz Biotechnology and applied at a dilution of 1:100 in PBS as primary antibody and incubated overnight. Texas Red conjugated goat anti-rabbit IgG (1:100, Jackson ImmunoResearch Labs) and FITC-conjugated rabbit anti-goat antibodies (1:100, BD Pharmingen) were applied and incubated for 30 minutes at room temperature.

### Data analysis

In our study, the Spearman's rank correlation between *Snail* and *VDR* was computed, and its statistical significance was assessed. A nonparametric approach was used in this assay. Gene expression data distribution was normalized by transformation by log_10_ for statistical analysis. In all statistical tests, two-tailed *p* values of .05 or less were considered statistically significant.

## Results

### VDR protein and gene expression levels in GHS rat intestine and kidney

Semiquantitative immunoblotting demonstrates a significant increase in VDR protein levels in GHS rat duodenum and kidney (1*A*), as reported previously.(31) VDR protein levels were elevated 9.9-fold in jejunum and 6.2-fold in ileum from GHS compared with NC rats (see 1*A*). In GHS rats, elevated VDR protein levels were comparable across duodenum, jejunum, ileum, and kidney cortex.

**Fig. 1:**
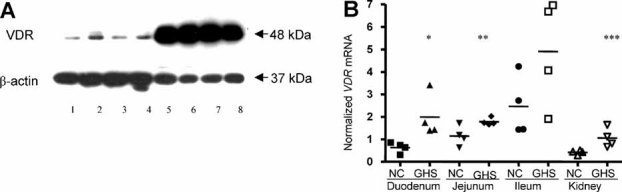
VDR is highly expressed in GHS rat intestine and kidney at the protein and mRNA levels. (*A*) Immunoblot for VDR and β-actin in total nuclear protein extracted from intestinal and kidney tissue from GHS and NC rats. Each lane represents intestinal or kidney extracts from an individual rat. All samples were run on the same blot. Lanes 1 to 4 are from NC rats; lanes 5 to 8 are from GHS rats. Lanes 1 and 5 are duodenum; lanes 2 and 6 are jejunum; lanes 3 and 7 are ileum, and lanes 4 and 8 are kidney. (*B*) Intestine and kidney *VDR* and *GAPDH* mRNAs from GHS and NC rats were subjected to real-time PCR using the primers in Table 1. *VDR* expression levels were normalized to *GAPDH* expression. *VDR* mRNA levels are increased in GHS versus NC rat duodenum, jejunum, and kidney. Results are individual values with mean shown as horizontal bars (*n* = 4 per group). *p = .027; **p = .034; ***p = .036.

Using primers for *VDR* (see Table 1), real-time PCR confirmed the previously reported increase in *VDR* mRNA in GHS rat kidney cortex(34) (see 1*B*). *VDR* mRNA was statistically significantly increased in GHS rat duodenal and jejunal but not ileal mucosa (see 1*B*). *VDR* mRNA expression levels varied by intestinal segment and were lower in kidney compared with all three regions of the small intestine analyzed. Duodenal *VDR* mRNA levels were increased 3.1-fold in GHS rats, whereas VDR protein levels were increased 6.3-fold. In GHS rat kidney, *VDR* mRNA and protein levels were raised 2.5- and 5.5-fold, respectively. The greater magnitude increase in VDR protein compared with mRNA in GHS rat duodenum and kidney supports previous observations(31) that increased VDR levels may be due to factors in addition to transcriptional regulation, including altered efficiency of *VDR* translational events and the stability of the VDR protein. Indeed, prolonged half-life of the GHS rat duodenal and renal cortical VDR protein was described in a previous study conducted in vivo.(31)

### Sequencing the GHS rat VDR exons, intron/exon boundary regions, and proximal promoter region

Samples of kidney genomic DNA from four GHS and two NC rats were subjected to DNA sequencing (data not shown). The results revealed no mutation, polymorphism, or splicing variant in the 5' UTR exons, CDS exons, intron/exon boundary regions, or 3' UTR exons of the *VDR.* There was no difference in the DNA sequence of the proximal 2 kb of the *VDR* proximal promoter region between the two groups. Therefore, the high mRNA levels of *VDR* in GHS rats may not result from mutation or allelic variation in the cDNA or proximal promoter regions. However, the studies do not exclude the possible regulation of *VDR* expression by an enhancer located far upstream, downstream, or within an intron.

### Snail expression is downregulated in GHS rats

To assess potential trans-regulation of *VDR* expression in GHS rats, mRNA analysis of *Snail* was performed. Immunoreactive Snail protein was faintly detectable by Western blot using whole-cell extract (data not shown) and was readily detectable in nuclear extracts from GHS and NC rats (2*A*), with a marked decrease in protein in GHS rat duodenal mucosa. Snail protein was faintly detectable in nuclear extracts of renal cortical tissue (see 2*B*) from NC but not GHS rats.

**Fig. 2:**
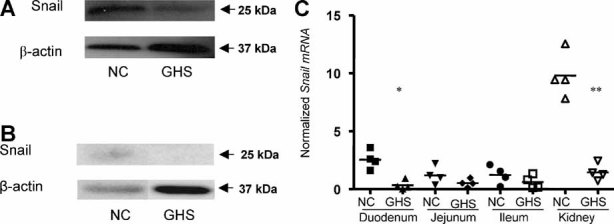
Snail is decreased in GHS rats at the protein and mRNA levels. (*A*, *B*) Immunoblots for Snail and β-actin of nuclear protein extracts from duodenum and kidney from individual GHS and NC rats. Representative results from an individual GHS and NC rat are shown. Snail protein levels were decreased in (*A*) duodenum and (*B*) kidney. (*C*) Total RNA isolated from intestinal and kidney tissue from GHS and NC rats was subjected to real-time PCR using primers listed in Table 1. *Snail* mRNA levels in GHS rat duodenum (*p = .026) and kidney (**p = .0002) were decreased (*n* = 4). Each data point represents intestinal or kidney extracts from a single rat. *Snail* levels in jejunum and ileum were not statistically significantly different between GHS and NC rats.

**Fig. 3:**
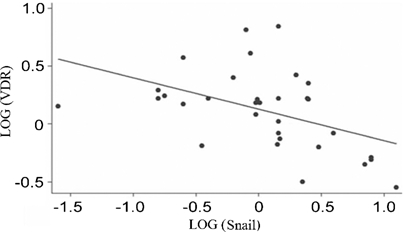
*Snail* mRNA is inversely correlated with *VDR* mRNA. Total RNA isolated from GHS and NC rat intestine and kidney was subjected to real-time PCR using the primers listed in Table 1. Expression levels of *VDR* and *Snail* mRNA were normalized to *GAPDH* mRNA expression. Each data point represents both *Snail* and *VDR* measured on the same tissue sample from each of 31 rats. The Spearman rank correlation coefficient of the individual measurements reveals a significant inverse relationship (*r* = –0.507, *p* = .0031). The regression line *y* = 0.128 – 0.271*x* has an intercept of 0.055, and the SE of the slope is 0.095.

In GHS rats, *Snail* mRNA levels were suppressed 7.5-fold in duodenum, 2.2-fold in jejunum, 2.0-fold in ileum, and 6.7-fold in kidney, with only the duodenal and kidney suppressions being statistically significant (see 2*C*). When measurements of *Snail* and *VDR* mRNA levels from the same tissue obtained from individual GHS and control rats were compared, there was a significant inverse relationship between *Snail* and *VDR* expression (Fig. 3). *Snail* and *VDR* expressions were inversely correlated by the Spearman rank correlation coefficient, where *r* = –0.507 and *p* = .0031.

### Snail binds to the E-box 3 within the rat VDR proximal promoter region

Wild-type mouse *Snail* was cloned into mammalian vector pcDNA3 (pcDNA3-*Snail*) and then transiently transfected into HEK293 cells. Nuclear extracts from the HEK293 cells transfected with pcDNA3-*Snail* contained a greater increase in Snail protein after 48 hours than cells that were transfected with the pcDNA3 empty vector (4*A*). EMSA and antibody-inhibition assays were performed using nuclear extracts from the transfected HEK293 cells. In the EMSA assay, the unlabeled rat E-box 1 (see 4*B*) and rat E-box 2 (see 4*C*) probes did not compete with the labeled probes for binding to the DNA-protein complexes. These results indicate that Snail does not bind specifically to the rat E-box 1 and 2 sequences. However, a specific DNA-protein binding complex was demonstrated when the rat E-box 3 DNA probe was incubated with pcDNA-*Snail-*transfected nuclear extracts (see 4*D*). When excess nonlabeled E-box 3 oligonucleotides were added to the incubation, the DNA-protein binding complexes were reduced. Furthermore, anti-Snail antibody inhibited the Snail DNA-protein binding, whereas the control using normal IgG was without effect (see 4*D*). These results indicate that Snail specifically binds to rat E-box 3. The EMSA data suggest that Snail could regulate *VDR* transcription through binding to the E-box 3 site located within the *VDR* promoter.

**Fig. 4:**
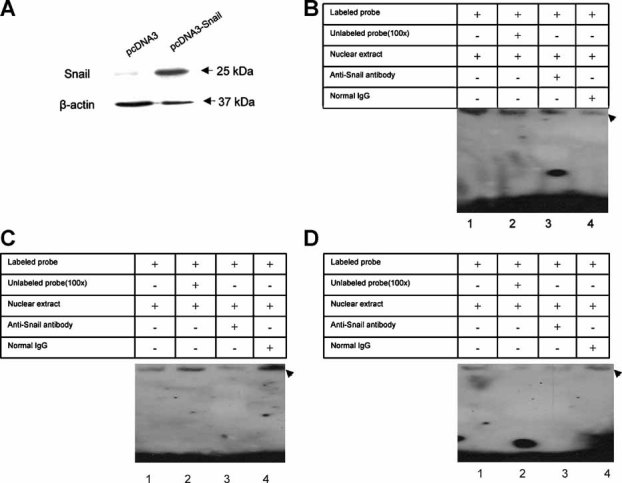
Snail protein binds to E-box 3 within the rat *VDR* promoter. (*A*) Immunoblots of Snail and β-actin were performed 48 hours following transient transfection of HEK293 cells with vectors containing either the empty control (pcDNA3) or the *Snail* gene (pcDNA3-Snail). EMSA shows the binding of Snail to oligonucleotides containing sequences for E-box 1 (*B*), E-box 2 (*C*), and E-box 3 (*D*). Unlabeled oligonucleotides were used as competitors. In panels *B*, *C*, and *D*, lane 1 shows the binding of labeled E-box probe with pcDNA3-Snail nuclear extract; lane 2 shows unlabeled oligonucleotides competing with labeled oligonucleotide probes for binding to the DNA-protein complex; lane 3 shows DNA-protein complex formation in the presence of Snail antibody; and lane 4 shows lack of inhibition of the DNA-protein complex by normal IgG. Note that Snail does not specifically bind to either E-box 1 (*B*) or E-box 2 (*C*) sequences but does bind specifically to E-box 3 sequence (*D*).

### Snail binds to the VDR promoter in vivo

Snail and VDR mRNA and protein levels in HEK293, DLD1, and SW480 cells were measured by real-time PCR (5*A*) and Western blot (5*B*). *VDR* mRNA levels were significantly higher and *Snail* mRNA levels significantly lower in DLD1 and SW480 cells compared with HEK293 cells (see 5*A*). VDR protein levels also were higher in DLD1 and SW480 cells than in HEK293 cells (see 5*B*), and Snail protein levels were lower in DLD1 cells than in HEK293 and SW480 cells (see 5*B*). The statistically significant differences in *Snail* and *VDR* mRNA levels across the three cell lines with higher *VDR* levels associated with lower *Snail* levels strongly suggest that the inverse relationship between Snail and VDR levels found in the tissues from GHS and NC rats (Figs. 1 through 3) also may exist within the cell lines examined. Based on the evidence that Snail binds to the human *VDR* promoter in vivo,(37) we used the three human cell lines (noted earlier) that varied in VDR content to explore Snail binding to the *VDR* promoter. Endogenous Snail occupied the *VDR* promoter in SW480 and HEK293 cells, but no Snail binding was detected in DLD1 cells (see 5*C*), in which *Snail* expression was very low (see 5*A*). The primers used to amplify the *VDR* promoter sequence included the E-boxes. Therefore, our results strongly suggest that Snail regulates *VDR* expression through binding to the *VDR* promoter in vivo.

**Fig. 5:**
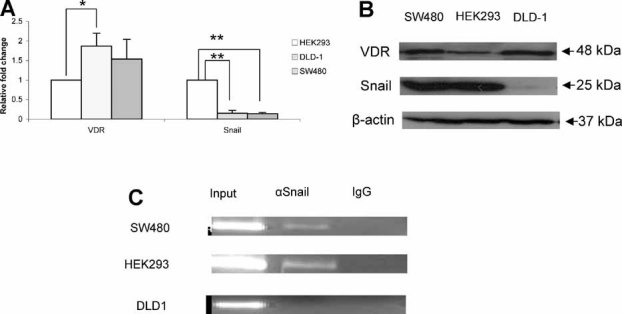
Snail binds to the *VDR* proximal promoter in vivo. (*A*) RNA extracted from HEK293, DLD1, and SW480 cells was subjected to real-time RT-PCR using specific primers for VDR, Snail, and β-actin. The relative expression levels of VDR and Snail in HEK293 were assigned a value of 1.0. Values are mean ± SEM for an *n* = 4 per experimental group. *p < .05; **p < .001. (*B*) Nuclear extracts from HEK293, DLD1, and SW480 cells were subjected to immunoblot using antibodies against VDR, Snail, and β-actin. β-actin was used as an internal control. (*C*) ChIP assays were performed on extracts of SW480, HEK293, and DLD1 cells using anti-Snail antibody. Normal IgG was used as a negative control. DNA was precipitated with either anti-Snail antibody or normal IgG. The primer pair used to amplify the human *VDR* promoter is described in “Materials and Methods.” Shown is a representative amplification of the input, DNA precipitated by the anti-Snail antibody, and normal IgG. The ChIP assay was repeated at least twice for each cell line.

### Hyperacetylation of histone H_3_ around GHS rat VDR promoter

*Snail* gene expression and protein levels are downregulated in GHS rat intestine and kidney (see Fig. 2). Tissue ChIP assays revealed Snail binding to the *VDR* promoter region close to E-boxes in intestine of NC rats, with weaker binding in GHS rat intestine (6*A*). Hyperacetylation of histone H_3_ was observed in the same *VDR* promoter region in intestine and kidney of GHS rats (6*B*).

**Fig. 6:**
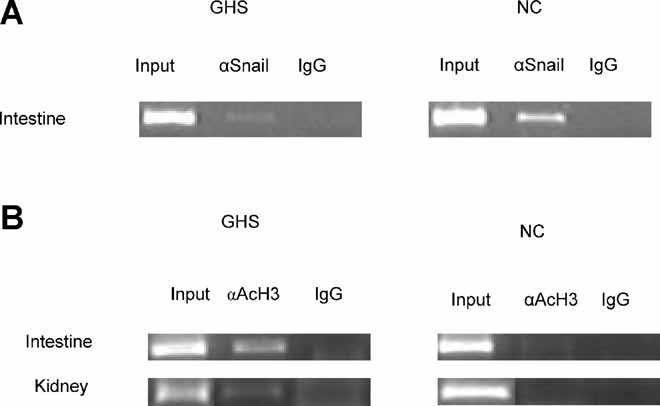
Hyperacetylation of histone H_3_ (AcH_3_) around the rat *VDR* promoter region in GHS rats. (*A*) ChIP assay on chromatin extracted from intestine of GHS and NC rats using anti-Snail antibody. The normal IgG was the negative control. Note the greater level of Snail detected in nuclear extracts of intestinal cells from NC rats. (*B*) AcH_3_ was detected in chromatin extracts from intestine and kidney from GHS and NC rats by ChIP assays using anti-AcH_3_ antibody. The PCR products of input and DNA when precipitated with anti-AcH_3_ antibody or normal IgG using the same primers as in panel *A* revealed greater acetylation of histone H_3_ in the GHS rat *VDR* promoter. The tissue ChIP assays were repeated at least twice for each sample of intestine and kidney tissue.

### Coexpression of VDR and Snail in small intestinal epithelial cells of GHS rats

*VDR* is expressed in intestinal villus cells (7*A, C*) and crypt cells (7*D, F*) and localizes within the epithelial nuclei. *Snail* has the same expression pattern within the intestinal epithelia and colocalizes with VDR in the same nuclei (see 7*B, C, E, F*)

**Fig. 7:**
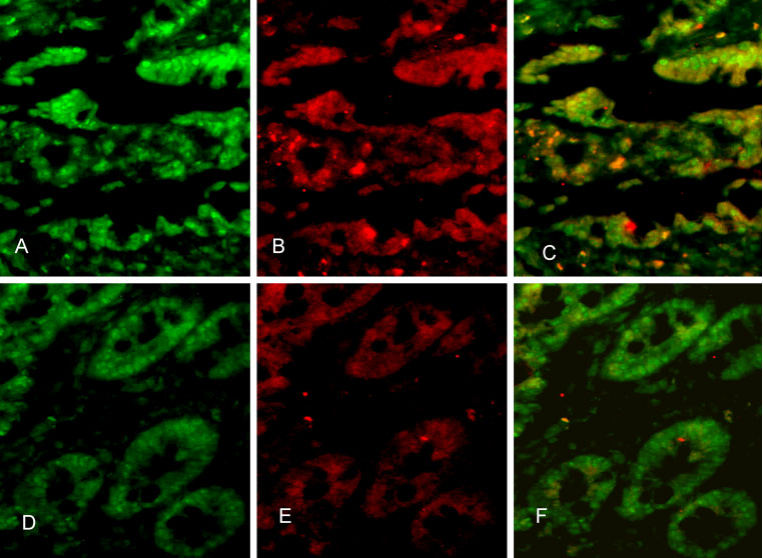
Immunolocalization of intestinal VDR and Snail. VDR (*A*, *D*) and Snail (*B*, *E*) were coexpressed (*C*, *F*) in nuclei of small intestinal epithelial cells. VDR was expressed in a greater epithelial population and with higher signal intensity than Snail. Fluorescence staining of VDR and Snail revealed that both were coexpressed in the nuclei of cells composing the intestinal villi (*A–C*) and crypt glands (*D–F*).

### Localization of VDR and Snail in the loop of Henle and distal convoluted tubules in GHS rats

**Fig. 8:**
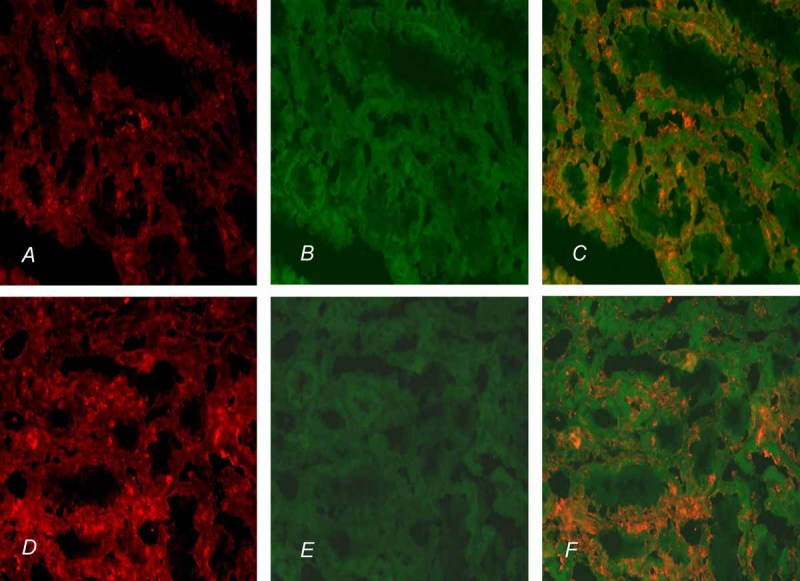
Immunolocalization by fluorescence staining of renal VDR and Snail. VDR (*A*, *D*) and Snail (*B*, *E*) were coexpressed (*C*, *F*) in renal epithelial cells of the loop of Henle (*A–C*) and the distal convoluted tubule (*D–F*). VDR was expressed in a greater epithelial population and with higher signal intensity than Snail.

Fluorescence staining of VDR and Snail revealed that both were expressed in the epithelial cells composing the loop of Henle (Fig. 8*A–C*) and distal convoluted tubule (Fig. 8*D–F*). Furthermore, VDR (see 8*A, D*) and Snail (see 8*B, E*) were coexpressed (see 8*C, F*) in the same renal epithelial cells in both nephron segments. VDR was expressed in a greater epithelial population and with higher signal intensity than Snail.

## Discussion

In GHS rats, hypercalciuria results from simultaneous increases in intestinal Ca absorption(16) and bone resorption(17) and decreased tubule Ca reabsorption.(18) Under physiologic and pharmacologic conditions, 1,25(OH)_2_D_3_ increases intestinal Ca transport, increases urine Ca excretion, and stimulates osteoclast-mediated bone resorption.(45,46) However, serum 1,25(OH)_2_D levels are normal and not elevated in GHS rats, and therefore, excess circulating 1,25(OH)_2_D cannot account for the increases in Ca transport that results in hypercalciuria. An alternative hypothesis that elevated tissue VDR levels may amplify the biologic effects of normal serum 1,25(OH)_2_D levels was confirmed in GHS rats by demonstrating elevated VDR levels in intestine,(16) bone,(17) kidney,(18) and splenic monocytes (personal communication). Elevated intestinal calbindin 9 kDa(31,47) renal calbindin 28 kDa(31,47) and renal Ca-sensing receptor(34) gene expressions and protein levels support the hypothesis that elevated tissue VDR levels are functional and serve to amplify the biologic actions of normal circulating 1,25(OH)_2_D levels through increased expression of vitamin D–dependent genes whose protein products regulate cellular Ca transport.

This study explores potential mechanisms that may elevate VDR levels in GHS rat tissues. In previous studies in GHS rats, the in vivo half-life of VDR protein and *VDR* mRNA in duodenum and kidney were observed to be prolonged,(31) and this may be responsible for at least a portion of the elevated VDR protein and *VDR* mRNA levels. In this study, semiquantitative real-time PCR detected several-fold increases in *VDR* mRNA levels in GHS rat duodenal and renal cortical tissue (see 1*B*). Regional increases in intestinal *VDR* mRNA levels also were noted, with elevated levels in duodenum and jejunum. Thus elevated tissue VDR levels can result from both increased *VDR* gene expression and protein synthesis and prolongation of VDR protein and mRNA half-life.(31) The relative contributions of extended VDR half-life and increased *VDR* gene expression to total VDR protein tissue levels have not been studied specifically. However, it is worth noting that in this study GHS rat duodenal *VDR* gene expression was increased 3.1-fold compared with control (NC) rats, whereas VDR protein levels were increased 6.3-fold. The level of duodenal VDR protein in this study was greater than reported in studies using earlier generations of GHS rats (two- to fourfold increase),(21,32) suggesting that the progressive increase in tissue VDR content with time may be due in part to increased *VDR* gene expression.

The mechanism whereby *VDR* gene expression is increased in GHS rats was further investigated by *VDR* gene DNA sequencing. Previous reports(47) of normal cDNA sequence of the *VDR* mRNA and normal migration of the VDR protein by Western blot suggested that a mutation or allelic variation of the *VDR* gene likely was not responsible for *VDR* gene overexpression in GHS rats. In this study, DNA sequencing of the intron/exon boundaries and the 5' flanking promoter region of the *VDR* gene in GHS rats failed to demonstrate any DNA sequence differences between the GHS rats and wild-type controls. Further, no allelic variation or mutation was identified that could account for the observed upregulation in *VDR* gene expression. A recent study(48) indicates that 1,25(OH)_2_D_3_ autoregulates mouse *VDR* genes expression through binding to three enhancer sites located 20 to 29 kb downstream of the transcriptional start site and within the *VDR* gene. Since we did not sequence the introns of the *VDR* gene in this study, we have not excluded the possibility of an enhancer-sequence variation in one or more introns that may increase the transcription of *VDR* in GHS rats.

In the absence of evidence for a mutation or allelic variation of *VDR* in GHS rats, we considered a dysregulation of a normal *VDR* gene. To this end, a recent study of human colon cancer tissues(37) in which repression of *VDR* gene expression was accompanied by overexpression of the *Snail* gene raised the possibility of an inverse relationship between *VDR* and *Snail* gene expression in GHS rats. Snail is a zinc-finger transcription factor that is expressed in migratory processes during embryonic development(38) and has been implicated recently in the phenotypic changes in colon cancer cells from an epithelial to a mesodermal morphology as the cells take on metastatic potential.(39,40) In the colon cancer cells, upregulated *Snail* is inversely correlated with cellular dedifferentiation and low *VDR* expression. In this study, GHS rat tissues we examined had high *VDR* gene expression that was accompanied by repression of *Snail* gene expression. Thus the increased VDR tissue levels appear to be due to elevated gene expression of a normal *VDR* gene, and the elevated *VDR* gene expression is inversely related to suppression of the *Snail* gene. The mechanism whereby suppression of Snail could enhance *VDR* expression centered around the interaction of Snail and E-box binding sites within the *VDR* promoter.(49)

The rat *VDR* promoter is highly analogous to the human *VDR* promoter (Table 3), with particular high homology of the DNA sequences of the E-boxes and their flanking regions. The consensus binding site for *Snail*-related genes contains a core of six bases (CAGGTG)(37) with a sequence motif that is identical to the E-box, which is the consensus core-binding site of the basic helix-loop-helix (bHLH) transcription factors.(50) The sequence indicates that Snail protein might compete with the bHLH factors for the same binding sequence. Indeed, Palmer and colleagues(37) demonstrated that Snail protein binding to E-boxes within the human *VDR* promoter was associated with decreased *VDR* gene expression. Mutation of the E-box that inhibited Snail binding also prevented repression of *VDR* gene expression. In the human *VDR* promoter there was high-affinity binding of Snail to each of three oligonucleotides containing each of the core E-boxes in the *VDR* promoter.(37) In contrast, the three E-boxes within the GHS rat *VDR* promoter demonstrated high specific binding of Snail protein at the rat E-box 3 but not at E-boxes 1 and 2. The specific binding pattern in the GHS rat *VDR* promoter may be due to conserved DNA sequence of E-box 3 but not 1 and 2 (see Table 3). The data suggest that the E-box 3 is a putative binding site for transcriptional repression by Snail of *VDR* gene expression. Recently, another example of transrepression by binding to E-box-type elements in promoters of some negatively regulated target genes for *VDR* was described.(51) The recent observations suggest that E-box binding may serve as a site of transrepression that is mediated by one or more repression factors in addition to Snail.(49) In the GHS rats, we postulate that suppression of Snail removes tonic repression of *VDR* and permits its overexpression. The cause of reduced *Snail* expression in GHS rat tissues is not known because this study was not designed to investigate the mechanism through which Snail is suppressed.

**Table 3:** Sequence Homology Between Human and Rat *VDR* Promoter Regions

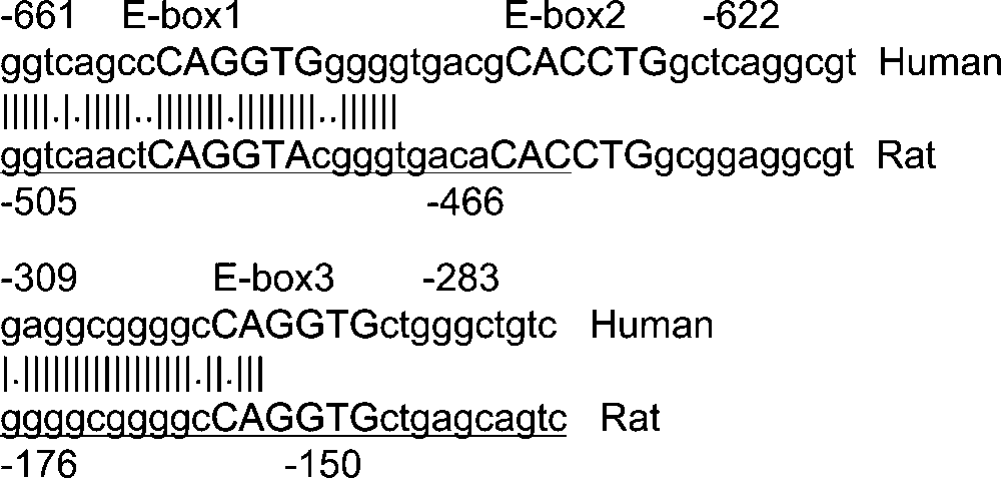

This study also explored potential mechanisms whereby suppressed Snail in GHS rats results in *VDR* overexpression. We found endogenous Snail binding to the *VDR* promoter in stable human colon and kidney cell lines that contain VDR (see 5*C*), which provides evidence that Snail regulates *VDR* expression. More important, we found Snail binding to the *VDR* promoter in NC but weaker in GHS rats (see 6*A*). These results indicate that under the pathologic condition in GHS rats, lower levels of Snail reduced repression of *VDR* expression. Furthermore, greater acetylation of histone H_3_ around the rat *VDR* promoter region in GHS rat intestine and kidney indicates that in GHS rat tissues (see 6*B*), the chromatin structure within the *VDR* promoter is more architecturally favorable to promote transcription. Therefore, the in vivo experiments indicate that the higher tissue VDR in GHS rats is associated with lower *Snail* expression and the subsequent epigenetic changes in the *VDR* promoter. Further, immunostaining showed coexpression of nuclear VDR and Snail within intestinal mucosal cells (see Fig. 7) and loop of Henle and distal convoluted renal epithelial cells (see Fig. 8), indicating that Snail can downregulate *VDR* expression at the same time and intracellular location. The colocalization of VDR and Snail within the nuclei of cells composing the loop of Henle and the distal convoluted tubule support a direct role for VDR and Snail in regulating renal Ca transport.

In conclusion, in GHS rats, elevated VDR levels can mediate all the observed changes in VDR target-tissue Ca transport. Snail exerts its suppressive effects on *VDR* expression through binding to one or more E-boxes within the proximal *VDR* promoter. Therefore, in GHS rats, decreased *Snail* expression lessens its suppressive actions and may result in *VDR* upregulation. To our knowledge, this is the first example of dysregulation of the *Snail* gene in a noncancerous genetic disorder.
